# Origin and development of two Escherichia coli clones vertically transferred in broiler production

**DOI:** 10.1099/mgen.0.001516

**Published:** 2025-11-28

**Authors:** Yufei Zhao, Annet Heuvelink, John Elmerdahl Olsen, Louise Poulsen, Henrik Christensen

**Affiliations:** 1 Department of Veterinary and Animal Sciences, University of Copenhagen, Copenhagen, Denmark; 2 GD Deventer, Deventer, Netherlands

**Keywords:** colibacillosis, colicin virulence (ColV), Grantham distance, most recent common ancestor, pan-genome, phage tail protein

## Abstract

Investigation of clonal development of dominant persistent clones of avian pathogenic *Escherichia coli* (APEC) is important to understand their evolution and to gain knowledge to improve their control in poultry production. Whole-genomic sequencing, including hybrid assembled genomes of short and long reads, was used to analyse clonal persistence and evolution of APEC. Two vertically transferred *E. coli* clones, represented by ten isolates from sequence type (ST) 95-PFGE type 65 and eight isolates from ST131-PFGE type 47, were selected to identify genomic variations. The isolates had been sampled in broiler production during a period of 9 months in a previous study. The main differences among strains within each clone were related to plasmids, transposases, incomplete phage elements and amino acid substitutions which by far exceeded the genetic variation related to core-genome SNPs (cgSNPs). Fourier-transform infrared spectroscopy was, for the most part, only able to trace clones within the same ST. The genome-wide mutation rate was equivalent to 1.48 mutations per genome per year for ST95-PFGE65 and 2.86 for ST131-PFGE47, respectively. The most recent common ancestors were estimated back to 2009 for ST95-PFGE65 and to 2011 for ST131-PFGE47, with further divergence occurring in years until sampling in 2014–2015. The methodology introduced is able to trace the temporal origin of APEC clones. The conventional threshold of ten or fewer cgSNPs to include strains in the same clone did not consider any gain or loss of plasmids for the strains compared. On average, one plasmid transfer event was predicted every second year. For strains expected to be vertically transferred during the long production periods of great-grandparents over grandparents and parents to broilers, one to two plasmid transfers are therefore predicted, and several cgSNPs may be introduced, whereas up to one cgSNP is expected to be manifested during a broiler production cycle and rarely involving plasmid transfer.

**Impact Statement**

Methodology was introduced to trace the temporal origin of avian pathogenic *Escherichia coli* (APEC) clones aiming for analysis of transmission which may contribute to prevention and control of APEC. Diversity in bacteriophage tail proteins, transposons and sequence divergence among proteins was observed for strains considered to belong to the same clone.

## Data Summary

The authors confirm that all supporting data, code and protocols have been provided within the article or through supplementary data files. Sequence data are available in the NCBI under assembly accession numbers JBELNB–JBELNS01, JBMPPS01–JBMPPZ01 and CP160104–CP160129. Illumina reads are deposited in the BioProject PRJNA1123689 and Oxford Nanopore Technologies reads in PRJNA1123745. The code used for bioinformatics has been deposited in GitHub: https://github.com/Yufzha/APEC_clone_2025.

## Introduction

Avian pathogenic *Escherichia coli* (APEC) is the cause of various disease manifestations in poultry collectively referred to as colibacillosis [1]. The production of chicken meat and table eggs is increasingly affected by diseases caused by APEC, compromising economy, as well as animal health and welfare [2].

APEC is a subtype of extraintestinal pathogenic *E. coli* (ExPEC), and ExPEC may belong to different sequence types (ST) [3]; however, within the APEC subtype, ST95 and ST131 are the predominant STs, which can be isolated both from animal and human infections [4,5]. The expression of antimicrobial resistance genes (ARGs) and virulence-associated genes (VAGs) may be responsible for the pathogenicity of APEC including its zoonotic potential [1]. While a pool of VAGs has been identified to be of particular importance in APEC [6], it has neither been possible to identify a specific combination of VAGs indicative of APEC, or to establish positive correlations between the number of VAGs and the virulence of APEC strains [7]. Several studies have documented a possibility for APEC to accumulate VAGs and ARGs genes through horizontal gene transmission [1,8], and the colicin virulence (ColV)-like plasmids harboured in APEC have been reported to carry several VAGs that encode adhesins, toxins, iron sequestration and serum survival capabilities, mainly responsible for the causation of extraintestinal disease [9,11]. The ColV-like plasmids were estimated to have been acquired by ST95 in the eighteenth century and by ST131 at least six times since the 1940s within a specific sub-clone [12,13].

It is important to trace clones of APEC to identify the sources of vertical and horizontal transmission of colibacillosis. Clonal stability of APEC has been observed in the poultry production environment over the years, as well as during vertical transfer of APEC from mother hen to chicken [8,14, 15]. However, it has not been investigated how outbreak clones change over time once established in a production system. We have found slightly increased mutation rates in isolates from chronic infections in chicken [16], which indicated that the mutation rates may play a role in the adaptation of APEC to long-term persistence in the infected host. Unfortunately, the timeframe for such changes is not precisely known.

It is important to investigate mutational changes at the clonal level since frequent genetic changes may invalidate analysis of source tracing. On a narrow time schedule, it is also interesting to investigate if clones which are vertically transferred in production systems may take up certain VAG and/or genes encoding for ARGs during the production period. In the past, PFGE had been the preferred method for short-term investigations of genetic relatedness, and MLST had been the method for more global surveillance of APEC [2]. Dramatic technological advancements have now made whole-genome sequencing the most powerful tool to investigate clones of pathogenic bacteria in regional outbreaks, as well as in the surveillance of VAGs and ARGs, and with reduced costs, this method is now feasible for everyday epidemiological investigations [17,18].

Accurate whole genomic sequences (WGSs) are needed to trace clones and maybe, more importantly, to detect mutations in space and time. The Illumina sequencing platforms have been widely used for the last two decades. They can generate high-accuracy (≥99.99%) raw reads with a relatively low cost [19] and enable extraction of information of MLST, prediction of VAGs and ARGs and clonal comparison by SNP analysis [2]. Nevertheless, the paired-end raw reads generated are often no longer than 250 to 300 bp, which makes it difficult to circularize a bacterial genome, leaving only incomplete contiguous sequences (contigs). The fragmented contigs may hinder the determination of whether VAGs and ARGs are located on chromosomes or mobile genetic elements. Another problem is the lack of knowledge of contig orientation and of structural repetitive regions and larger rearrangement events such as transposon, insertion sequence and genetic cassette activity, which are larger than the sequencing length of the Illumina reads [19,20]. The long-read sequencing platforms, such as Pacific Biosciences and Oxford Nanopore Technologies (ONT), can generate single DNA reads with a median length of 10 kbp and up to 100 kbp or longer, depending on the setup [19,21], and thus can be a great improvement to correct sequences generated with Illumina technology with respect to the repetitive and other complex regions within genomes [22]. Although the raw read accuracy can meet 99.5% with the new R10.4.1 flow cell and V14 kit according to the ONT website, hybrid-assembled genomes with a combination of the accuracy of short-read sequencing and the completeness of long-read sequencing still provide the most reliable and cost-effective approach to reconstruct bacterial genomes [23,24].

The aim was to investigate clonal development of dominant clones of APEC during persistence in the broiler production system, to observe the genetic events and to determine the hypotheses for the evolution of the clones.

## Methods

### Sample collection and molecular typing

The strains investigated in the current study were isolated from cloacal swabs in hatcheries, broilers affected by yolk sac infection or septicaemia, and from cases of salpingitis (inflammation of the fallopian tubes) in four broiler breeder farms (denoted farms 722, 723, 729 and 730) with normal mortality, where 40% (*n*=71/178) and 23% (*n*=41/178) of the *E. coli* salpingitis infections were related to isolates belonging to APEC of ST95 and ST131, respectively [14]. The four broiler breeder farms had, in pairs (722+723; 729+730), received layers from the same rearing flock. For the current investigation, strains of one PFGE type [25] of ST95 (ST95-PFGE65) and one PFGE type [26] of ST131 (ST131-PFGE47) collected during a 9-month period in 2014 and 2015 were carefully selected to represent broiler parents, newly hatched chicks and broilers affected by disease (Table 1).

**Table 1.** Strains of *E. coli* isolated and reported by Poulsen *et al.* [14] with associated ST and PFGE type which were further investigated by whole-genome sequencing. INSDC accession numbers of assembled whole genomic sequences are listed in Table S5

Strain	Origin	Organ/lesion	ST	PFGE	Clade†
729-270514-x-12*	Broiler parent	Salpingitis	95	65	1.1
729-140714-2-2	Broiler	Yolk sac infection	95	65	1.1
723-121214-2-6	Broiler	Liver/septicaemia	95	65	1.1
729-080814-2-9	Broiler parent	Salpingitis	95	65	1.2
729-180914-36s	Hatchery	Cloacal swab	95	65	1.2
729-021014-1-4	Broiler parent	Salpingitis	95	65	1.2
729-xx-1-3-yolk	Broiler	Yolk sac infection	95	65	1.2
722-111214-3-1	Broiler	Liver/septicaemia	95	65	1.2
729-070215-1-6	Broiler	Yolk sac infection	95	65	1.2
730-281114-27s	Hatchery	Cloacal swab	95	65	2
729-240514-x-10	Broiler parent	Salpingitis	131	47	1
729-141114-1-15	Broiler parent	Salpingitis	131	47	1
723-150514-43n	Hatchery	Cloacal swab	131	47	2
723-220514-1-6	Broiler	Liver/septicaemia	131	47	2
722-270514-2-6	Broiler parent	Salpingitis	131	47	2
722-020714-2-3	Broiler parent	Salpingitis	131	47	2
723-250714-31n	Hatchery	Cloacal swab	131	47	2
723-010814-2-1	Broiler parent	Salpingitis	131	47	2

*(Broiler breeder farm – day-month-year – animal – isolate from the animal). For hatcher isolates, n refers to normal eggs and s to soiled eggs.

**Fig. 1.**
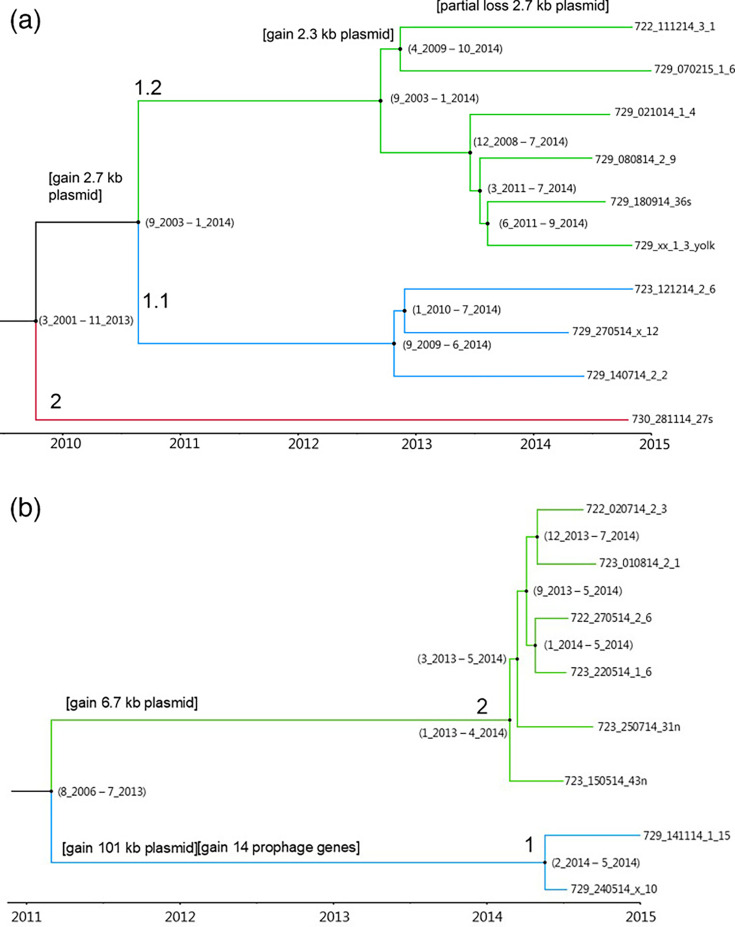
Time-calibrated maximum credibility trees inferred by BEAST. The X-axis represents the BEAST-estimated emergence time (year). A total of 79 non-recombinant SNPs were used to build the tree for ST95-PFGE65 (**a**), in which green branches refer to subclade 1.2, blue branches refer to subclade 1.1 and the red branch refers to clade 2. A total of 43 non-recombinant SNPs were used to build the tree for ST131-PFGE47 (**b**), in which green branches refer to clade 2 and blue branches refer to clade 1. The 95% highest posterior density (HPD) CIs of time estimates of month and year are shown in parentheses at nodes.

†Clade numbers and colours resulting from phylogenetic analysis (Fig. 1 and Fig. S1, available in the online Supplementary Material)

### DNA extraction

*E. coli* isolates were cultured from stocks stored frozen in glycerol at −80 °C. Cultivation was initiated on blood agar (Tryptose Blood Agar Base, Difco, Brøndby, Denmark) added with 5% bovine blood with subsequent incubation at 37 °C for 24 h. A single colony from each plate with pure and abundant growth of *E. coli* was cultured in Luria–Bertani broth at 37 °C overnight. The DNA was extracted with Maxwell RSC Cultured Cells DNA kit (Promega, Madison, USA) according to the manufacturer’s instructions. Later, the Quick-DNA HMW MagBead Kit (Zymo Research) was used for Nanopore sequencing since DNA quality based on OD260/230 was not acceptable for ST131 strains extracted by the Maxwell RSC Cultured Cells DNA kit. The purity and concentration of extracted DNA were evaluated by a NanoDrop spectrophotometer (Thermo Fisher Scientific) and a Qubit fluorometer (Thermo Fisher Scientific). The integrity of DNA was determined by gel electrophoresis. DNA extracted from the isolates was used for both Illumina and ONT sequencing.

### Library preparation and sequencing

DNA Libraries for Illumina sequencing were prepared using Illumina DNA Prep (Illumina, San Diego, CA), and sequence reactions were prepared with Miseq Reagent kit v2 (Illumina, San Diego, CA). Samples of ST95-PFGE65 (*n*=10) and ST131-PFGE47 (*n*=8) (Table S1) were sequenced by the Illumina MiSeq platform using a 2×250 bp paired-end sequencing strategy. In parallel, strains from each clone were sequenced by ONT on a MinION Mk1B sequencer (ONT, Oxford). Libraries of selected DNA samples were prepared by using Rapid Barcoding Kit 96 V14 (SQK-RBK114, ONT) and were loaded on one MinION Flow Cell (R.10.4.1 FLO-MIN114, ONT) for sequencing for 48 h with an accurate model (260 bp).

MinKNOW software v5.4.3 was used to perform the sequencing and data acquisition. Post-run base-calling and demultiplexing were performed with Dorado v0.5.3. The base-calling model was dna_r10.4.1_e8.2_400bps_sup@v4.2.0.

### Raw read quality control and trimming

Quality control, adapter removing and short reads filtering of Illumina reads were performed on fastp v0.12.4 for Linux with default parameter settings [27]. For ONT long reads, the short and low-quality reads were filtered by Filtlong v0.2.1 (https://github.com/rrwick/Filtlong) with the settings filtlong --min_length 1000 --keep_percent 95. Quality control of long reads was assessed using SeqKit v2.5.1 [28].

### Genome assembly and annotation

SPAdes v3.13.1 was used with a ‘--careful’ parameter for genome assemblies [29] for short reads generated by Illumina. Contig sizes smaller than 1,000 bp were removed from the assembled genomes. CLC Genomic Workbench 22 (QIAGEN) was used for short-read assemblies to compare the assembly quality with SPAdes. Hybrid-assembled genomes were completed by two workflows: Unicycler v0.5.0 was used directly with Illumina short reads and corresponding ONT long reads [21,30]. Quast v5.0.2 [31] and BUSCO v5.5.0 [32] were used to evaluate the quality of assemblies. Annotation was performed with Prokka v1.4.0 with ‘--force --centre X –compliant’ parameters [33]. The visualization of assembly quality was performed with the ggplot2 package in R (version 4.3.1).

### Pan-genomic and phylogenomic analysis

Roary v3.12.0 [34] was used to identify the core genome and accessory genome among the two APEC clones with an option of ‘-e –n –p 20 –f –r’. The gene presence and absence matrix table generated from Roary was further used for annotation comparison.

The SNP approach was performed to reconstruct the phylogeny and further infer a Bayesian time-scale phylogenetic tree of two APEC clones. Snippy v4.6.0 (https://github.com/tseemann/snippy) was used for variant calling and alignment of the core genome. For ST95-PFGE65, WT strains were mapped onto the *E. coli* reference APEC O1 genome (accession no. CP000468.1). Gubbins v3.3.1 [35] was used to detect and mask recombination regions within the core genome alignment.

The SNPs of recombinant-free core-genome alignments of the two clones [core-genome SNP (cgSNP)] were extracted by SNP-sites v2.5.1 [36]. IQ-Tree v2.2.2.6 [37] was used to infer maximum likelihood trees for non-recombinant cgSNP alignments with the GTR (general time-reversible)+G model and bootstraps of 1,000 repetitions.

### Bayesian temporal analysis

To locate the most recent common ancestor (MRCA) for ST95-PFGE65 and ST131-PFGE47, 79 non-recombinant cgSNPs in ST95-PFGE65 and 43 non-recombinant cgSNPs in ST131-PFGE47 were used for Bayesian temporal analysis. Linear regression analysis was performed in TempEst v1.5.3 (https://github.com/beast-dev/Tempest/releases) to estimate the strength of the temporal signal between the root-to-tip genetic distances extracted from recombination-free maximum likelihood trees. Bayesian inference of non-recombinant cgSNPs in the two clones was performed in BEAST v2.7.6 [38] based on the GTR substitution model. Multiple combinations of molecular clock models (strict and optimized relax clock model) and population size change models (constant population, exponential growth population and Bayesian skyline) were tested. For each of these six combinations, three independent replicates of Markov chain Monte Carlo generations were conducted for 100 million generations and sampled every 1,000 steps for convergence. The quality of all combinations was assessed in Tracer v1.7.2 (http://github.com/beast-dev/tracer/), of which a desirable result should present effective sample sizes of all parameters more than 200. The outcome of the analysis was to use the strict molecular clock model and the exponential growth population model as best-fitting models. LogCombiner (in the BEAST2 package) was used to combine replicated analyses for the chosen model combinations with removal of 10% burn-in. Afterwards, maximum clade credibility (MCC) trees were generated by TreeAnnotator (in the BEAST2 package). The MCC trees were finally visualized and edited in FigTree v1.4.4 (https://github.com/rambaut/figtree).

### Prediction of AMR and VAG genes, types of fimbria and ST131 clades

ResFinder (v4.3.3; software version: 08/22/2023; database version: 04/12/2023) [39] was used to identify acquired ARG genes. PointFinder (software version: 08/22/2023; database version: 05/03/2023 (http://genepi.food.dtu.dk/resfinder) was used to detect chromosomal point mutations. The tools were used with default parameters (threshold identity ≥90%; minimum length ≥60%). VirulenceFinder (v2.0.3; database version: 12/02/2022) [40] was used to identify the VAGs of *E. coli* genomes. A custom dataset containing 46 genes associated with APEC virulence and fitness features [41] was downloaded from https://github.com/JohnsonSingerLab/APEC_VF_database and used for a blastn search against the APEC dataset when there were gaps between virulence identification and dataset. Further interpretation of virulence-associated genes was obtained based on the review of Ovi *et al*. [6]. Fimbria types were predicted with FimTyper 1.0 (https://cge.food.dtu.dk/services/FimTyper/) [42]. For ST131 strains, clade affiliation was based on the reference sequences reported by Kallonen *et al*. [43]. Clade affiliation was controlled by average nucleotide identity [44] determined on EzBioCloud (https://www.ezbiocloud.net/). The search for colibactin-coding regions was done by antiSMASH [45].

### Plasmid and prophage prediction and comparative analysis

Identification of plasmids was performed using PlasmidFinder (v2.0.1; software version: 07/01/2022; database version: 01/18/2023 with 95% minimum identity and 60% minimum coverage [46]. MOB-suite v1.4.9 was used to further type and reconstruct smaller plasmids [47]. The 'mob-recon' function in MOB-suite was used to identify if an incomplete contig belonged to the chromosome or plasmid. ‘Circular=True’ was marked at the title of each contig in the original Unicycler assembly if true. Multiple genome alignments were performed with MAUVE [48].

Evolutionary and functional differences between amino acid substitutions were evaluated using PAM (point accepted mutation) matrix 2 and the Grantham distance matrix [26,49]. Prophage regions were predicted with PHASTEST (https://phastest.ca) and serotype predictions by SerotypeFinder 2.0 [50]. Errors with Prokka annotations were removed according to Zhao *et al*. [51].

### Fourier-transform infrared spectroscopy

Fourier-transform infrared spectroscopy (FT-IR) analysis was performed using the IR Biotyper^®^ system (Bruker Daltonics GmbH & Co. KG, Bremen, Germany) as described by Landman *et al.* [52]. One suspension per strain was made, and then, four technical replicates were tested.

## Results

### Statistics of WGS and assemblies

The quality of short-read sequencing is summarized in Table S1 including short reads obtained for 18 strains and 14 of these strains which were also sequenced by long-read sequencing using ONT (Table S2). Around 1–2% of the genome length was missing from the short-read assemblies compared to the hybrid-assembled genomes (Table S2).

### Pan-genome and phylogenetic analysis

The two vertically transferred *E. coli* clones, each with the same PFGE type including ten isolates from ST95-PFGE65 and eight isolates from ST131-PFGE47, were selected to identify genomic variations based on short-read sequence assemblies. The isolates represented periods of sampling of 8 and 7 months for ST95-PFGE65 and ST131-PFGE47, respectively (Table 1). Pan-genomic analysis indicated that for the ST95-PFGE65 clone, the number of total genes was 5,235, in which 5,170 core genes (98.8%) were shared by all 10 isolates. For the ST131-PFGE47 clone, a total of 4,771 genes were found, in which 4,742 core genes (99.4%) were shared by all 8 isolates. Maximum likelihood trees were built based on cgSNPs for ST95-PFGE65 and ST131-PFGE47, respectively. After multiple alignment and variant calling, a total of 1,104 SNPs were found in ST95-PFGE65, while 232 SNPs were found in ST131-PFGE47. Recombination detection and filtering removed 92.3% of ST95-PFGE65 cgSNPs and resulted in 79 cgSNPs, while 81.5% of ST131-PFGE47 cgSNPs were removed, and 43 cgSNPs were identified as non-recombinant cgSNPs. Phylogenetic analysis revealed 2 major clades for ST95-PFGE65, of which clade 1 had 9 strains with a cgSNP range of 5–30 and included subclades 1.1 and 1.2 (Fig. S1A and Table S3). Subclade 1.1 included 3 strains with cgSNPs ranging from 10 to 14, while the 6 strains in subclade 1.2 shared a maximum of 15 cgSNPs. Clade 2 was only represented by strain 730_281114_27S and had 29 to 36 cgSNPs to the other strains. Two major clades were identified in ST131-PFGE47, of which clade 1 had 2 strains with 4 cgSNPs and clade 2 had 6 strains with 2–8 cgSNPs, while 28–32 cgSNPs were found between the 2 clades (Fig. S1B and Table S4).

### Common ancestors and divergence times

Linear relationships between divergence times and genetic distances were confirmed in both ST95-PFGE65 (correlation coefficient=0.60, *R*^2^=0.36) and ST131-PFGE47 (correlation coefficient=0.79, *R*^2^=0.63) clones through root-to-tip regression analysis. Time-calibrated phylogenetic trees estimated that the MRCA of strains included from ST95-PFGE65 was from 2009 (Fig. 1a). While strain 730_281114_27S directly originated from MRCA as clade 2, clade 1 diverged into two subclades in 2010. These two subclades further diverged in 2012, and the last divergence was found in 2013 between 729_180914_36S and 729_xx_1_3_yolk. The genome-wide mutation rate of the ST95-PFGE65 clone was estimated to 2.87×10^−7^ SNPs per site per year, equivalent to 1.48 mutations per genome per year. The 95% HPD CIs of time estimates were 5–6 years around the mean for the deep parts of the trees and 2–3 years for the more recent parts of the tree.

For ST131-PFGE47, the MRCA was dated to 2011 for divergence of strains into clade 1 and clade 2 (Fig. 1B). Further divergence within the two clades was estimated to have occurred from 2014. The genome-wide mutation rate of ST131-PFGE47 was estimated as 7.33×10^−7^ SNPs per site per year, equivalent to 2.86 mutations per genome per year. The 95% HPD CIs of time estimates were less for this tree compared to the ST95-PFGE65 tree.

### Prediction of chromosomes and plasmids

Plasmids were predicted by PlasmidFinder and the MOB-suite program. In addition, circularization was predicted by Unicycler. All 7 strains of ST95 PFGE65 were predicted to possess a chromosome in length from 5,152 to 5,232 kb, and they were all predicted with a 194 kb ColV-like plasmid (Table S5). Replicons IncFIB and IncFIC(FII) were predicted in this plasmid, and it could be categorized as ColV since it was of the IncFI type and harboured *cvaABC* genes in addition to genes coding for iron acquisition and potential virulence factors (Table 2). This ColV-like plasmid had 100% identity and coverage with a plasmid deposited in NCBI [53] from South Korea (acc. no. CP126927). All seven strains of ST95-PFGE65 were predicted with a 6.7 kb Col156 type of plasmid. This plasmid had 99.79 and 100% identity and coverage, respectively, to a plasmid deposited from an ETEC strain isolated from a pig (acc. no. CP122739). Five strains out of the seven ST95-PFGE65 were predicted with a 2.7 kb plasmid and four with a 2.3 kb plasmid (Table S5). The 2.7 kb plasmid was most closely related to the ColRNAI (acc. no. AJ627566) replicon with 92% identity, and this plasmid had 99.79 and 100% identity and coverage, respectively, to a range of *E. coli* genomes deposited with NCBI. The 2.3 kb plasmid was related to the Col(MG828) (acc. no. NC_008486) replicon with 87% identity. It had high identity and coverage to genomes of *E. coli* and other species deposited with NCBI. The seven ST131-PFGE47 strains available with hybrid-assembled genomes were predicted with a chromosome of 4,866–4,888 kb, and they were predicted with a 201 kb plasmid (Table S5). The plasmid was predicted with markers IncFIA, IncFIB and IncFIC(FII), and it could be categorized as ColV since it was of the IncFI type and harboured *cvaABC* genes in addition to genes coding for iron acquisition and potential virulence (Table 2). This ColV-like plasmid was related to the one found in ST95-PFGE65 strains with 99.9% identity and 90% coverage. It was also found in several other *E. coli* genomes deposited with NCBI.

**Table 2.** Prediction of potential APEC virulence genes in two persistent and vertically transferred *E. coli* clones belonging to ST95-PFGE47 and ST131-PFGE65.

Category	Virulence genes	Strains	
		ST95-PFGE65	ST131-PFGE47
		*n*=7	*n*=7
Adhesion	*fimH*	C*	C
	*papA_F11*	C	C
	*papC*	C	C
Iron acquisition	*chuA*	C	C
	*etsC(AB)*	P	P
	*fyuA*	C	C
	*ireA*	C	nd
	*iroN (BCDE)*	P	P
	*irp2*	C	nd
	*iucC(ABD)*	P	P
	*iutA*	P	P
	*sitA(BCD)*	P	P
Invasion	*ibeA*	C	C
	*tia*	C	nd
Autotransporter	*tsh*	P	P
	*vat*	C	nd
Toxin	*cvaC(AB)*, *cvi*	P	P
	*hlyF*	P	P
Serum survival	*iss*	P	P
	*kpsMII_K1*	C	C
Others	*traT*	P	P
	*ompT*	C	C

* P, identified with plasmid location; C, identified with chromosomal location

nd, not detected.

Strain 729_141114_1_15 had an additional Incl1-l plasmid replicon of 101 kb (Table S5). The plasmid was also identified in strain 729_240514_x_10 but not in the strains located in clade 2 of Fig. 1(b), and it had weak homology to sequences in NCBI where only fragments matched other plasmids. It can be assumed that these strains gained the large 101 kb plasmid and an incomplete phage after their divergence from clade 1 (see Fig. 1b) in 2011–2012.

Comparison showed that the ColRNAI-related plasmids (2.7 kb in ST95 strains, 6.7 kb in ST131 strains) matched only some regions of this plasmid, which indicated that the IncI1-l plasmid did not share the backbone with the other two but was a completely different plasmid. A 6.7 kb plasmid was identified in five strains of ST131-PFGE47. It was of the ColRNAI type (acc. no. NC_002119) with 93% identity. This plasmid was only found in the two strains of clade 2 (Fig. 1b) and not in clade 1, which was confirmed by read mapping. The plasmid was unrelated to the 6.7 kb plasmid predicted in ST95-PFGE65 strains. The plasmid was also represented in NCBI, e.g. acc. no. CP149315. All strains of ST131-PFGE47 carried a 1.6 kb plasmid being closest related to the Col(MG828) replicon with 93% identity. The plasmid was unrelated to the 2.3 and 2.6 kb plasmids in ST95-PFGE65 strains. This small plasmid matched a deposited plasmid (CP045286) with 100% identity and coverage, respectively. Strains 722_111214_3_1, 729_180914_36s and 729_24_05_14_x_10 possessed seven fragments of between 1.2 and 76 kb which could not be identified as plasmids related to the lack of marker genes and the lack of predicted circularization. Further DNA sequencing, assembly and predictions will be needed to completely circularize these genomes.

### Prediction of prophages

Each of the strains of ST95-PFGE65 was predicted with eight intact prophages (acc. nos. NC_005056, NC_019716, NC_042057, NC_001416, NC_019723, NC_027398, NC_019445, NC_021857 or NC_003356; similarity from PHASTEST) on the chromosome and one incomplete phage (NC_049919, NC_049342, NC_011357, NC_049342 or NC_049924) harboured on the ColV-like 193 kb plasmid totaling 406–379 kb (7.0–7.5% of the genome). Chromosomes of strains of ST131-PFGE47 were predicted each to harbour three intact prophages (NC_028943, NC_004745 and NC_019522). Strains 729-141114-1-15 and 729_240514_x_10, in addition, had an incomplete prophage of 31.5 kb (NC_049451). Slight variations in the prediction of prophages were observed related to the statistical outcomes of the search with PHASTEST. In total, the genomes of strains of PGFE47-ST131 were predicted to include 2.1–2.6% of prophage DNA sequences. The accession numbers listed all relate to *Caudoviricetes* which are tailed bacteriophages [54].

### Comparison of phage tail proteins

Phage tail proteins were analysed since they are associated with recognition of the bacterial host. Phage tail proteins identified from the Prokka annotation were used to query proteins in NCBI (Table S6). A total of six phage tail proteins were identified to vary across strains of ST95-PFGE65 at the protein sequence level. Four of these predicted proteins only included single amino acid substitutions; however, in the other two, up to seven substitutions were found. For ORF 018010 of strain 729_021014_1_4, 43 amino acid substitutions were observed compared to strain 729_180914_36S. The phylogeny of homologues of ORF 01807 of strain 729_021014_1_4 is shown on Fig. S2. Two closely related homologues are present in all strains, both with inter and intra protein sequence variation, indicating that duplication of the underlying genes happened before the strains diverged (paralogues). Only three phage tail proteins were found to vary between strains of ST131-PFGE47; however, one included a difference in the length of the protein of up to 165 amino acids (Table S6). Variations in phage tail proteins followed the major clades identified by cgSNP phylogenetic analysis (Figs 1 and S1). Grantham distances between amino acid substitutions varied from 22 (e.g. FY) to 133 (e.g. NV) and PAM2 odds ratios from −1 (e.g. ED) to −6 (e.g. NV) with averages of Grantham distances of 26–81 and PAM2 odds ratios of –2 to −4.

### Prediction of AMR and VAG genes, fimbria and serotypes

According to ResFinder, the sulfamethoxazole resistance gene *sul2* was predicted at the same position and length in all genomes of ST95-PFGE65. The *sul2* gene was located on the chromosome in a 30 kbp region with ARGs and genes associated with mobile genetic elements. blastn search showed a similar region in an APEC strain from Korea (acc. no. CP126926), and part of the region was present in other *E. coli* and also other species. The manganese and iron uptake, as well as disinfection-resistant gene *sitABCD*, were predicted in hybrid-assembled genomes of all strains of both ST131-PFGE47 and ST95-PFGE65, where they were located on the chromosomes and large ColV-like plasmids (Table 2). VirulenceFinder predicted a total of 22 and 18 VAGs associated with single genes or operons in the hybrid-assembled genomes of ST95-PFGE65 and ST131-PFGE47, respectively (Table 2). In ST95-PFGE65, one of two *gad* genes and *ireA* was not predicted in short-read genomes but in all hybrid-assembled genomes, and *sitA* was not predicted in genomes generated from short-read sequencing of 729-270514-x-12 and 729_xx_1_3_yolk but in the hybrid-assembled genomes. In ST131-PFGE47, both of the two *gad* genes were not predicted in short-read genomes but in all hybrid-assembled genomes. The lack of one of the *gad* genes may be related to problems assembling repeated regions which are longer than Illumina short reads. These results indicate a chance of false-negative prediction of VAGs in genomes assembled from short reads only. Fimbria types were predicted as H526 and H22 for ST95-PFGE65 and ST131-PFGE47 strains, respectively. The genomes of ST95-PFGE65 were all predicted to be serotype O2/O50:H5, whereas the genomes of ST131-PFGE47 were predicted to be O25:H4. For strains of ST131, all strains belonged to clade B according to the reference sequences provided by Kallonen *et al.* [43]. The non-ribosomal peptide metallophore type I polyketide synthase island (*pks*) was predicted with medium confidence in ST95 strains by antiSMASH but not in ST131 strains. In addition, the *pks* gene region (acc. no. AM229678) was confirmed to be present in ST95 strains by a blast search. None of the potential virulence factors were located in the regions of the prophages predicted above.

### Comparison of predicted proteins

**Fig. 2.**
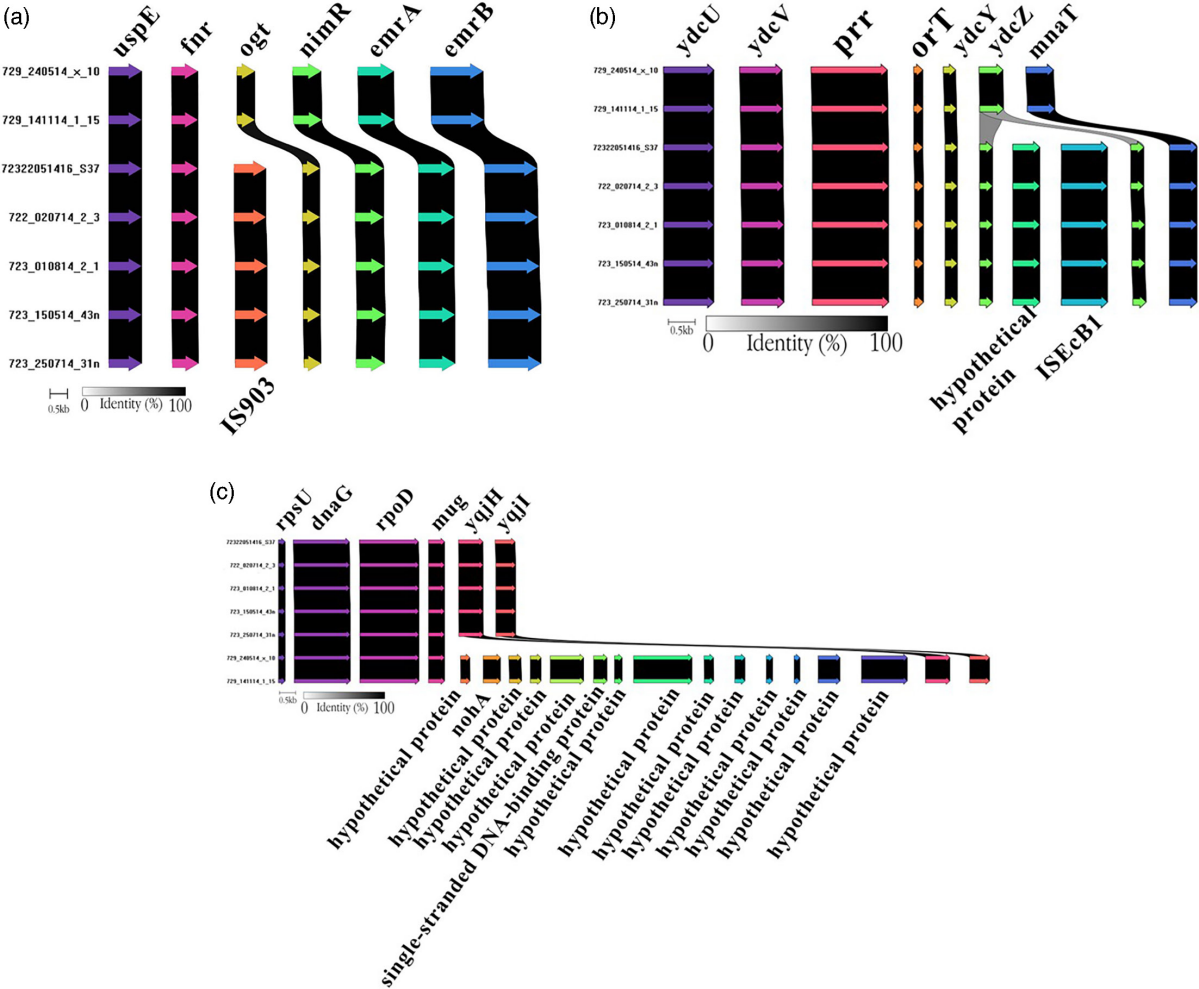
Genetic context of representative predicted genes and their flanking regions of ST131-PFGE47 strains with scaling. Differences between strains of the same clone were mainly caused by transposon insertions, such as (**a**) when an IS*5* family transposase IS*903* was inserted between flanking genes and (**b**) when an IS*3* family transposase IS*EcB1* was inserted into gene *ydcZ*. Another situation was found in (**c**), where a fragment including phage DNA-packing protein NohA encoded by *nohA* was only inserted in strain 729_141114_1_15.

Differences in gene content were found on few occasions among strains of the ST131-PFGE47 clone mainly related to transposon insertions, such as seen in Fig. 2(a) where an IS*5* family transposase IS*903* was inserted between flanking genes and in Fig. 2(b) where an IS*3* family transposase IS*EcB1* was inserted into gene *ydcZ*. Another situation is shown in Fig. 2(c), where a fragment including phage DNA-packing protein encoded by *nohA* was only inserted in strains 729_141114_1_15 and 729_240514_x_10.

### Comparison of predicted protein sequences

**Fig. 3.**
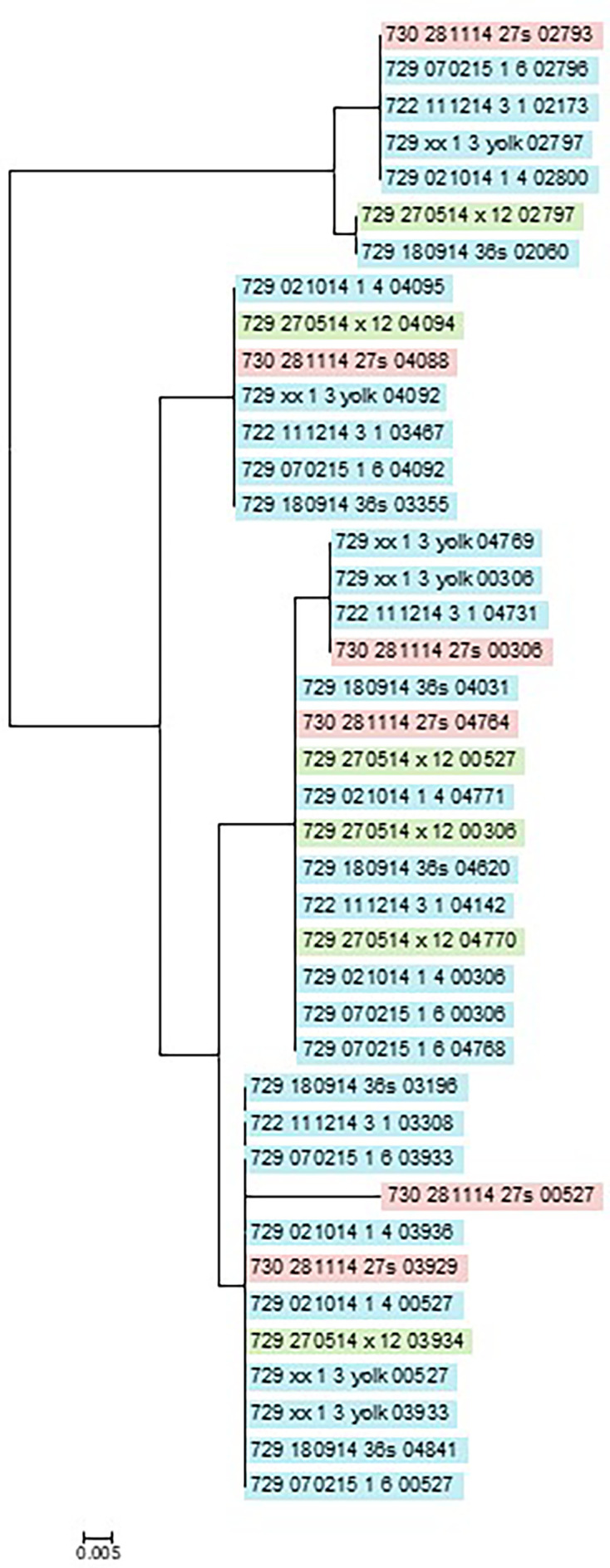
Phylogeny of predicted KlaC protein sequences present in multi-copy (ORFs 02800, 04095, 04771 and 03936 of strain 729_021014_1_4) of the ST95-PFGE65 clone showing paraphyletic relationships. The scale bar indicates sequence divergence given the substitution matrix (Jukes and Cantor) and algorithm for the tree (neighbour joining). Strains are coloured reflecting the clades in Figs 1 and Fig. S1.

Comparison between the hybrid assembled genomes using MAUVE and blast identified 36 and 39 predicted proteins with protein sequence differences between strains within the clones, ST95-PFGE65 and ST131-PFGE47, respectively (Tables S7 and S8). For ST95-PFGE65, 25 differences were in single-copy proteins, and the additional 11 differences were in proteins with high similarity both at intra and inter strain level. For ST131-PFGE47, only three of these intra and inter strain level differences were identified. These multiple differences were mainly identified in proteins annotated as transposons. The antirestriction protein KlcA was identified in six very similar versions both within and between strains (Fig. 3). The amino acid changes were rather uniformly distributed in the clades of ST95 strains, whereas there was a tendency for more substitutions between the two clades of ST131 strains. The phylogeny showed identical versions of the proteins in different strains and minor differences between homologues of each strain indicating that duplication of the underlying genes happened before the strains diverged (paralogues). In strains of ST131-PFGE47, a large outer membrane protein (ORF 01924 in 723_010814_2_1) annotated as EntS/YbdA MFS transporter diverged in length of two strains (723_010814_2_1 and 729_240514_x_10) with 97 and 293 amino acids compared to the other five strains (Table S8). However, this divergence did not follow the clades determined from the cgSNP phylogeny (Fig. 1b). The phylogeny of a hypothetical protein present in two copies followed a paraphyletic pattern of evolution (Fig. S3) in parallel to the examples shown for phage tail protein and KlcA sequences. Grantham distances between amino acid substitutions varied from 10 (e.g. IM) to 215 (e.g. CW) and PAM2 odds ratios from −1 (e.g. ED) to −11 (e.g. CW).

### FT-IR

**Fig. 4.**
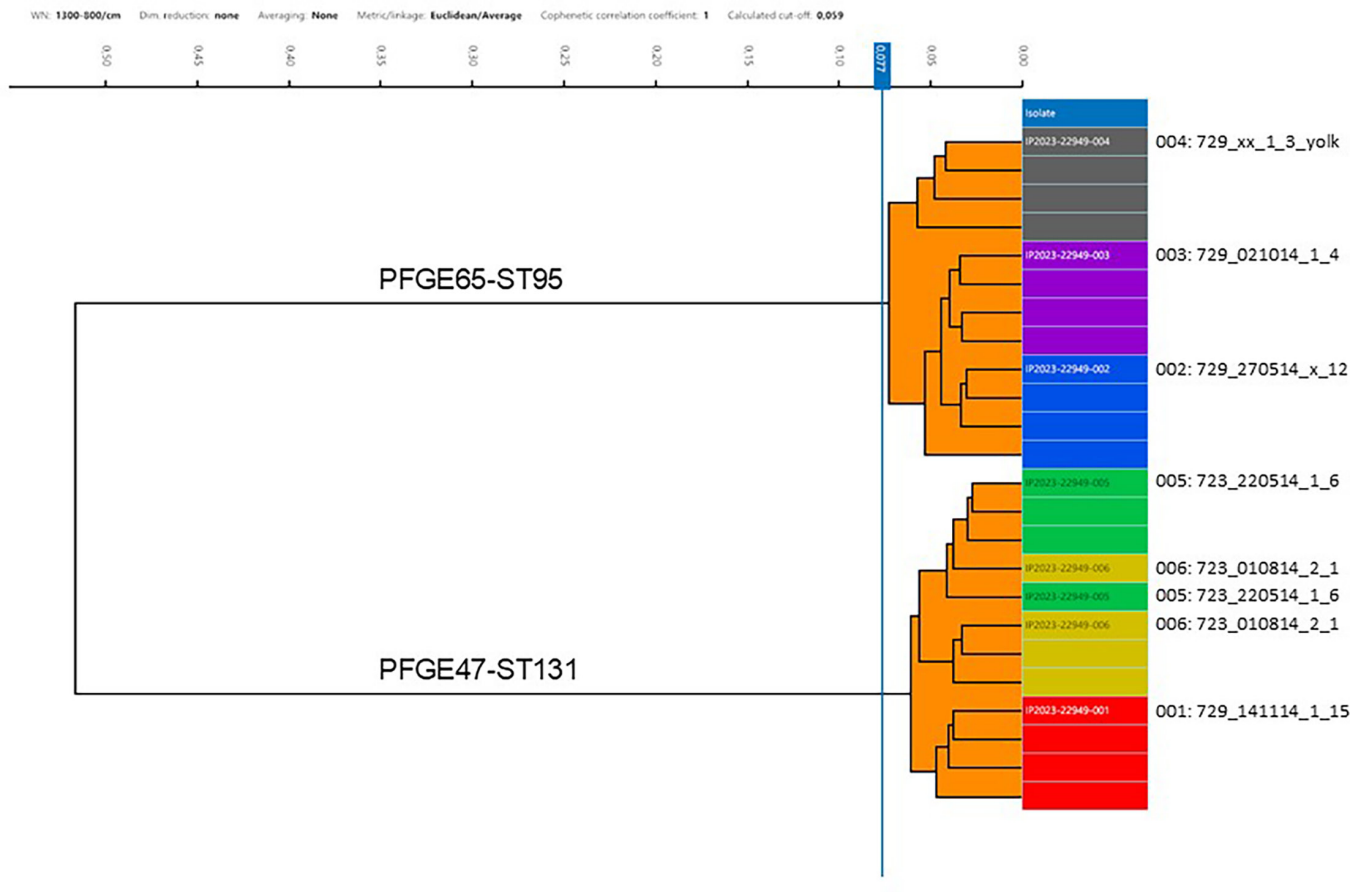
FT-IR analysis of six strains of two clones (ST95-PFGE65 and ST131-PFGE47) of APEC performed according to Landman *et al.* [52] with four replications of each strain.

Comparison of the six strains analysed by hybrid-assembled genomes showed two deep clusters corresponding to ST95-PFGE65 and ST131-PFGE47, respectively, clearly documenting the ability of FT-IR to separate STs at a cut-off of 0.077. In addition, all three ST95-PFGE65 strains were separated. For ST131-PFGE47, two strains, 723_220514_1_6 and 723_010814_2_1 (4 SNPs), could not be separated for all replicates, whereas the third, 729_141114_1_15, came out with a separate cluster for all replicates (Fig. 4).

## Discussion

ST95 and ST131 are among the four predominant STs which can be isolated from ‘colibacillosis’, others being ST23 and ST117 [2,55]. We chose to investigate ST95 and ST131 because of the strong zoonotic potential documented for clones of these STs [4,5]. The strains had been isolated from broilers and broiler parents that died in relation to normal mortality in conventional Danish broiler production. Conventional Danish broiler production has a high level of biosecurity which includes all-in-all-out management both for broilers and broiler parents, and horizontal transmission of bacteria is therefore less likely compared to vertical transmission [2,14]. In previous studies, it has been revealed by PFGE and MLST that some specific clones of pathogenic *E. coli* can be vertically transmitted from broiler parents to broilers and cause high mortality and morbidity [8,14, 56, 57]. Transmission is expected to happen both as genuine vertical transmission by infection of the embryo from the hen and as pseudo vertical transmission by contamination of the eggshell followed by colonization of the chick upon hatching. The current investigation used WGS to facilitate high-resolution analysis of clonal persistence and evolution of vertically transmitted APEC clones ST95-PFGE65 and ST131-PFGE47.

Hybrid-assembled genomes combining short and long reads are preferred to close the genomes by utilizing the long reads to close repeated sequences and, at the same time, increase accuracy with the benefit of the short reads [23,58]. More plasmids were identified in *E. coli* by use of this hybrid assembly approach [24]. In the current study, hybrid-assembled genomes allowed more important VAGs, ARGs, phage elements and plasmids to be predicted compared to the short-read assemblies. The serotype predictions of ST95-PFGE65 strains as O2/O50:H5 are in line with observations of Kravik *et al*. [59]. These strains were predicted to carry the colibactin set of virulence genes, never found in ST131. The fimbrial type H22 in ST131-PFGE47 is characteristic for the foodborne uropathogenic clone, and serotype O25:H4 is well known in clade B [12,43, 60].

Clade B of ST131 is one of the dominant clones well known from human bloodstream infections [61]. The genes *cva* and *cvi* encoding microcin V were predicted on the large ColV plasmids of both ST95 and ST131 strains (see Table 2). A recent study [13] showed that these plasmids with associated microcin V genes may be responsible for clonal success and why we observed these clones vertically transferred in poultry production from broiler parents and to broilers. It was shown by Arredondo-Alonso *et al*. [13] that strains of ST95 and ST131 with the microcin V genes were able to outcompete other *E. coli in vitro*. Further investigations of these mechanisms, both *in vitro* and *in vivo*, are highly encouraged to explain the success of certain APEC clones in poultry production.

cgSNP analysis is considered the most sensitive tool to trace outbreaks of pathogenic bacteria. For ST117, we have previously found 7–36 cgSNP differences of *E. coli* isolated from cellulitis over a period of 15 months [15]. Another investigation of the same ST117 outbreak in broilers found an average of 23 cgSNPs between 47 isolates from both diseased broilers and broiler parents from Scandinavia [57]. Bhattacharjee *et al*. [61] found epidemic isolates of *E. coli* ST131 isolated from neonatal septicaemia in the same tertiary care centre from the same year to have 20 cgSNPs. For paired urinary tract isolates of ST131 from the same patients sampled with at least a 30-day interval, 2–18 cgSNPs were found [62]. We may conclude that the number of up to 36 cgSNPs found in the current study is within the limits of *E. coli* clones with common ancestry reported in other studies. Within the limit of 36 cgSNPs that defined a clone, differences between strains of each clone were also related to plasmids, transposases and prophage elements.

Roer *et al*. [63] defined presumptive national outbreaks (PNOs) for ST131 isolates with a SNP distance below or equal to 10. Ludden *et al*. [64] identified zero to five SNPs between highly related isolates from the same animal species but sampled from different farms. Using the threshold of ten cgSNPs for the current study excluded any gain or loss of plasmids. With the average of 6 cgSNP differences per year calculated from Fig. 1, at most one cgSNP can be expected during the production period (1–35 days) of broilers. However, more cgSNPs may be realized during the life of broiler parents (1–450 days) and grandparents (1–450 days). At this level, changes in plasmid content may also be realized since such events were predicted to happen on average every second year (calculated from Fig. 1). Assuming that clones are vertically transferred all the way in the broiler breeder hierarchy from great-grandparents via grandparents and parents to broilers, plasmid transfer events are likely to occur during the full production cycle, and also a number of cgSNPs may have been introduced. Strains with a few cgSNP differences may still have non-synonymous amino acid changes in some proteins since some protein sequence differences are filtered out during cgSNP analysis. For some proteins, paraphyletic relationships were even found among strains of the same clone related to their common ancestry.

The evolutionary impact of the amino acid substitutions was evaluated by the Grantham [49] index which is related to physio-chemical properties of the amino acids and by PAM2 which is related to the statistical comparison of observed substitution frequencies of similar proteins in a database. Both indices showed high variations both within and among proteins (range 10–215 for Grantham and −1 to −11 for PAM2). The highest possible Grantham score is 215 between cysteine and tryptophan, indicating that these amino acids are the most divergent with respect to physio-chemical properties, and their odds ratio is −11 in the PAM2 matrix. Within these ranges, the differences observed between the amino acid substitutions in the current study showed a huge diversity. The nature of the proteins that were observed with amino acid substitutions between strains of clones is not well described in the literature, and it is difficult to evaluate the effect of the mutations on the function of these proteins. Further work may have practical application, for instance, to develop attenuated vaccines in parallel to work done with *Mycoplasma synoviae* (e.g. 65).

With respect to the phage tail proteins, almost the same divergence was observed with respect to physio-chemical properties and odd ratios of amino acid changes as for the chromosomal proteins, and also here, it is difficult to evaluate the effect of the mutations on the function of the proteins. Functional changes may only be observed during infection of new bacteria if the phages turn into lytic phase.

The genome-wide mutation rate of strains of ST95-PFGE65 of 2.87×10^−7^ SNPs per site per year was consistent with other reported *E. coli* mutation rates (4.39×10^−7^ [25]; 4.14×10^−7^ [66]). For ST131-PFGE47, 7.33×10^−7^ SNPs per site per year was also consistent with published temporal analysis of *E. coli* mutation rate (7.15×10^−7^ [67]; 6.0×10^−7^ [68]).

FT-IR is a good screening method since it saves money and can be used to select a subset for WGS, and it is able to separate isolates at the ST level, but the exact level of discrimination still has to be figured out. Based on the limited data from this study, we may conclude that discrimination only goes to the ST level. Using the threshold from above, then FT-IR is probably not able to identify PNO clones. Few investigations have documented the ability of FT-IR to identify clones of *E. coli* [69,70]; however, the relationship to cgSNP analysis is not well characterized. For routine investigation of strains of APEC, the use of FT-IR can be recommended as both faster and cheaper than WGS at the cost of some resolution. Further studies will have to be initiated to investigate if FT-IR can detect gain and loss of plasmids and other mobile genetic elements.

Genomic changes of *E. coli* observed may have been introduced by changes in the management of chicken production. In 2012, the British Poultry Council banned the use of third- and fourth-generation cephalosporins and recommended that fluoroquinolones should only be used as a last resort (https://britishpoultry.org.uk/wp-content/uploads/2024/08/BPC-2017-Antibiotics-Report-Web.pdf). In accordance with this, until 2010 and 2011, day-old grandparent chicks imported from Scotland to Sweden, which provide broiler parents and broilers in Denmark, were sampled for cephalosporin-resistant *E. coli* [56]. We speculate that the ban on the use of third- and fourth-generation cephalosporins and the use of fluoroquinolones as a last resort may have partially contributed to clone development since the clones investigated in the current study were without predicted acquired antibiotic resistance genes to cephalosporins and quinolones. Antibiotic resistance genes for sulphonamide were only predicted for ST95-PFGE65 strains and not for other types of antibiotics or in strains of ST131-PFGE47. This result is in accordance with the Danish surveillance for the year 2015 of samples from broilers and domestically produced meat that resulted in 56% of indicator *E. coli* isolates that were susceptible to all antimicrobials tested, whereas other isolates showed resistance towards ampicillin, tetracyclines, trimethoprim and sulphonamides [71].

## Conclusions and perspectives

The methodology introduced can trace the temporal origin of *E. coli* clones. A similar approach was used by Ludden *et al*. [64] for strains of ST117 in turkey production. This opens up a range of applications to prevent and control APEC and other pathogenic types of *E. coli* at specific points of origin. The 95% HPD CIs of time estimates may be improved by the analysis of more WGS from a more uniform time distribution. A precondition for this type of analysis is continuous archiving of strains with precise records of time of isolation. The clones defined by cgSNPs and the presence of the ColV-like plasmids indicated a narrow relationship among the strains compared. However, when the genomes of such strains were analysed, differences in plasmid content, bacteriophage elements, transposons and protein sequences indicated a higher degree of genetic diversity. The factors leading to genomic changes with respect to mobile genetic elements have not yet been incorporated into evolutionary models or studied in greater detail [72], and further research is needed into the mechanisms that promote relocation of mobile genetic elements of *E. coli* under both controlled laboratory and field conditions.

## Supplementary material

10.1099/mgen.0.001516 Uncited Supplementary Material 1.

## Abbreviations

APEC: avian pathogenic *Escherichia coli*
ARGs: antimicrobial resistance genes
cgSNPs: core-genome SNPs
ColV: colicin virulence
ExPEC: extraintestinal pathogenic *Escherichia coli*
FT-IR: Fourier-transform infrared spectroscopy
GTR: general time-reversible
HPD: highest posterior density
MCC: maximum clade credibility
MRCA: most recent common ancestor
PAM: point accepted mutation
PNOs: presumptive national outbreaks
VAGs: virulence-associated genes
WGSs: whole genomic sequences
