# Supplementary material for: Origin and development of two Escherichia coli clones vertically transferred in broiler production
Source: Microb Genom. 2025 Nov 28;11(11):001516. doi: 10.1099/mgen.0.001516 (PMC12662567; doi:10.1099/mgen.0.001516)
Supplement: Uncited Supplementary Material 1. [file mgen-11-01516-s001.pdf]

Supplementary information for the paper entitled:

Origin and development of two *Escherichia coli* clones vertically transferred in broiler production

By Yufei Zhao, Annet Heuvelink, John Elmerdahl Olsen, Louise Poulsen and Henrik Christensen

Legends to supplementary figures

Fig. S1 Maximum likelihood trees of clones ST95-PFGE65 (A) and ST131-PFGE47 (B) strains based on cgSNPs. The complete genome of APEC O1 was chosen as the reference. The scale bar here means the genetic distance/the number of substitutions per site between strains.

Fig. S2. Phylogeny of predicted phage tail proteins of duplicated genes (ORF 01807 and 02512 strain 729_021014_1_4) of the ST95-PFGE65 clone showing paraphyletic relationships. Strains are colored reflecting the clades in Fig. 1 and supplementary Fig. 1. The scale bar indicates sequence divergence given the substitution matrix (Jukes and Cantor) and algorithm for the tree (neighbour joining).

Fig. S3. Phylogeny of a predicted hypothetical and duplicated protein (ORFs 02527 and 03469 strain 723_010814_1_2) of the ST131-PFGE47 clone showing a paraphyletic relationship of the two homologs. Strains are colored reflecting the clades in Fig. 1 and supplementary Fig. 1. The scale bar indicates sequence divergence given the substitution matrix (Jukes and Cantor) and algorithm for the tree (neighbour joining).

References

Grantham R. Amino acid difference formula to help explain protein evolution. *Science* 1974; 185(4154):862-4. doi: 10.1126/science.185.4154.862.

Dayhoff MO, Schwartz RM, Orcutt BC. A model of evolutionary change in proteins, in Dayhoff MO. Atlas of Protein Sequence and Structure. *Natl Biomed Res Found*, Washington DC, 1978; 5(3): 345- 352.

Zhao Y, Olsen JE, Poulsen L, Christensen H. Comparison of genomic assembly and annotation based on two clones of avian pathogenic *Escherichia coli. bioRxiv* 2024.11.22.624809; doi: https://doi.org/10.1101/2024.11.22.624809

Fig. S1A

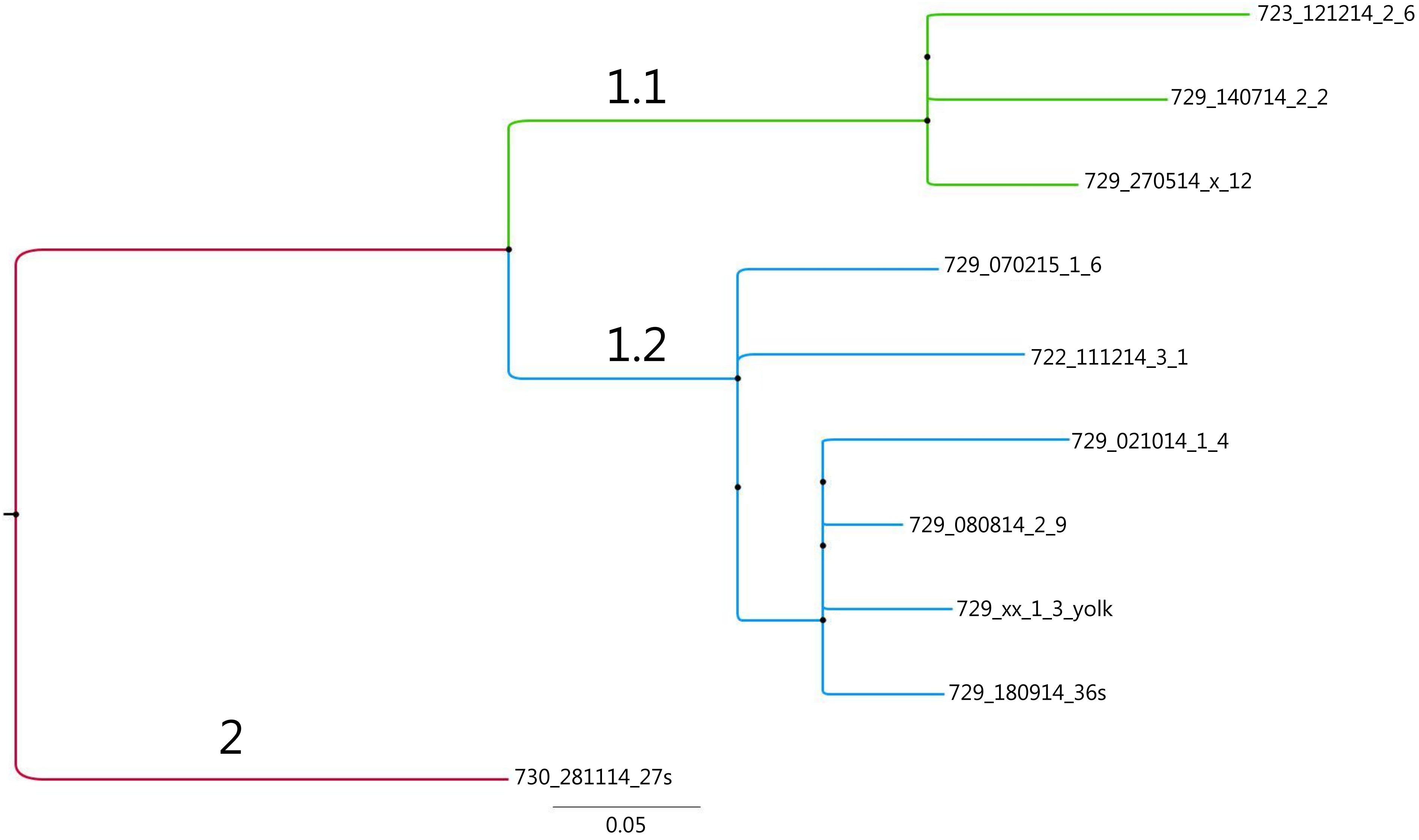

1.1
1.2
2
723_121214_2_6
729_140714_2_2
729_270514_x_12
729_070215_1_6
722_111214_3_1
729_021014_1_4
729_080814_2_9
729_xx_1_3_yolk
729_180914_36s
730_281114_27s
0.05

Fig. S1B

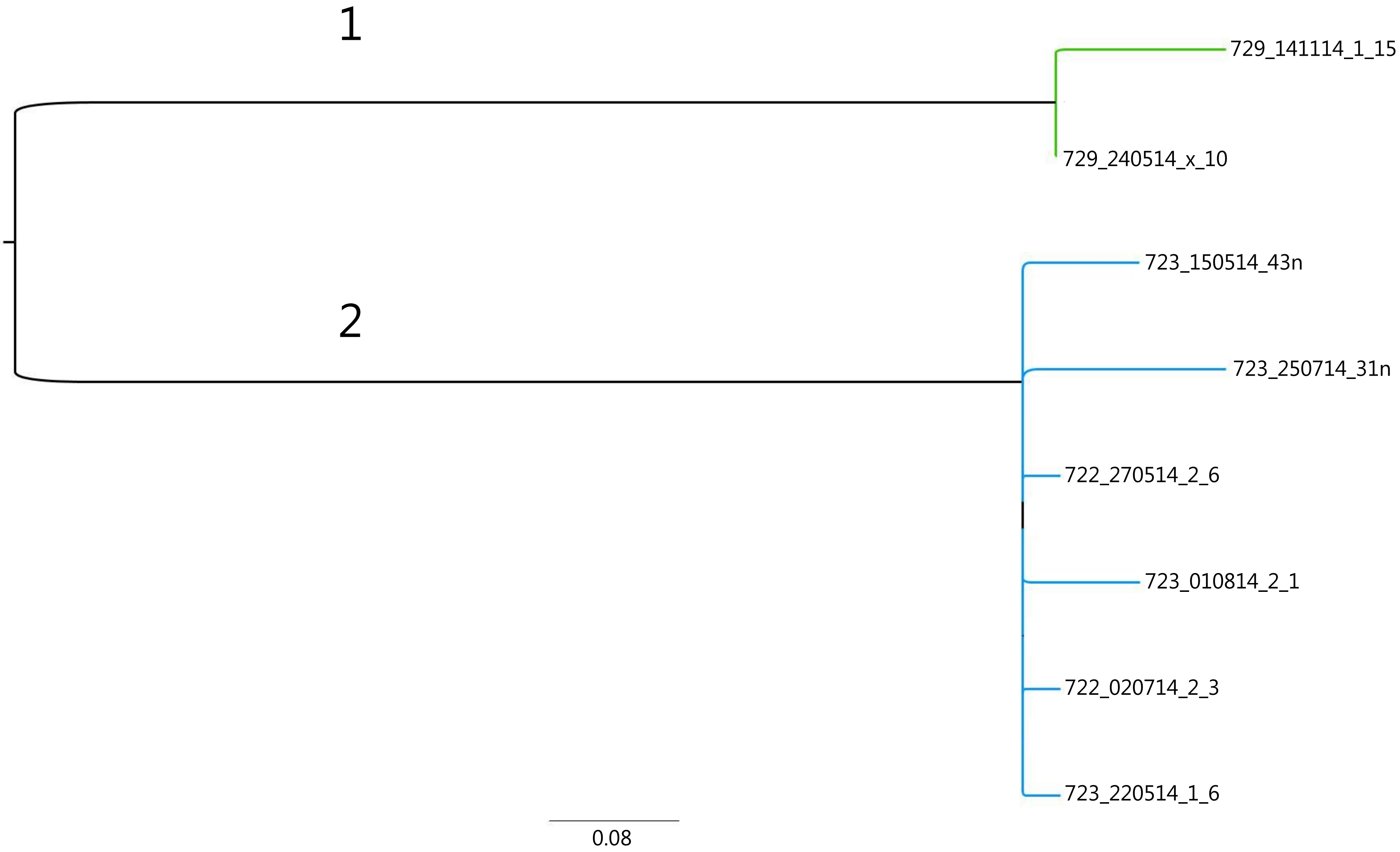
1
729_141114_1_15
729_240514_x_10
2
723_150514_43n
723_250714_31n
722_270514_2_6
723_010814_2_1
722_020714_2_3
723_220514_1_6
0.08

Fig. S2

ST95

Phage tail

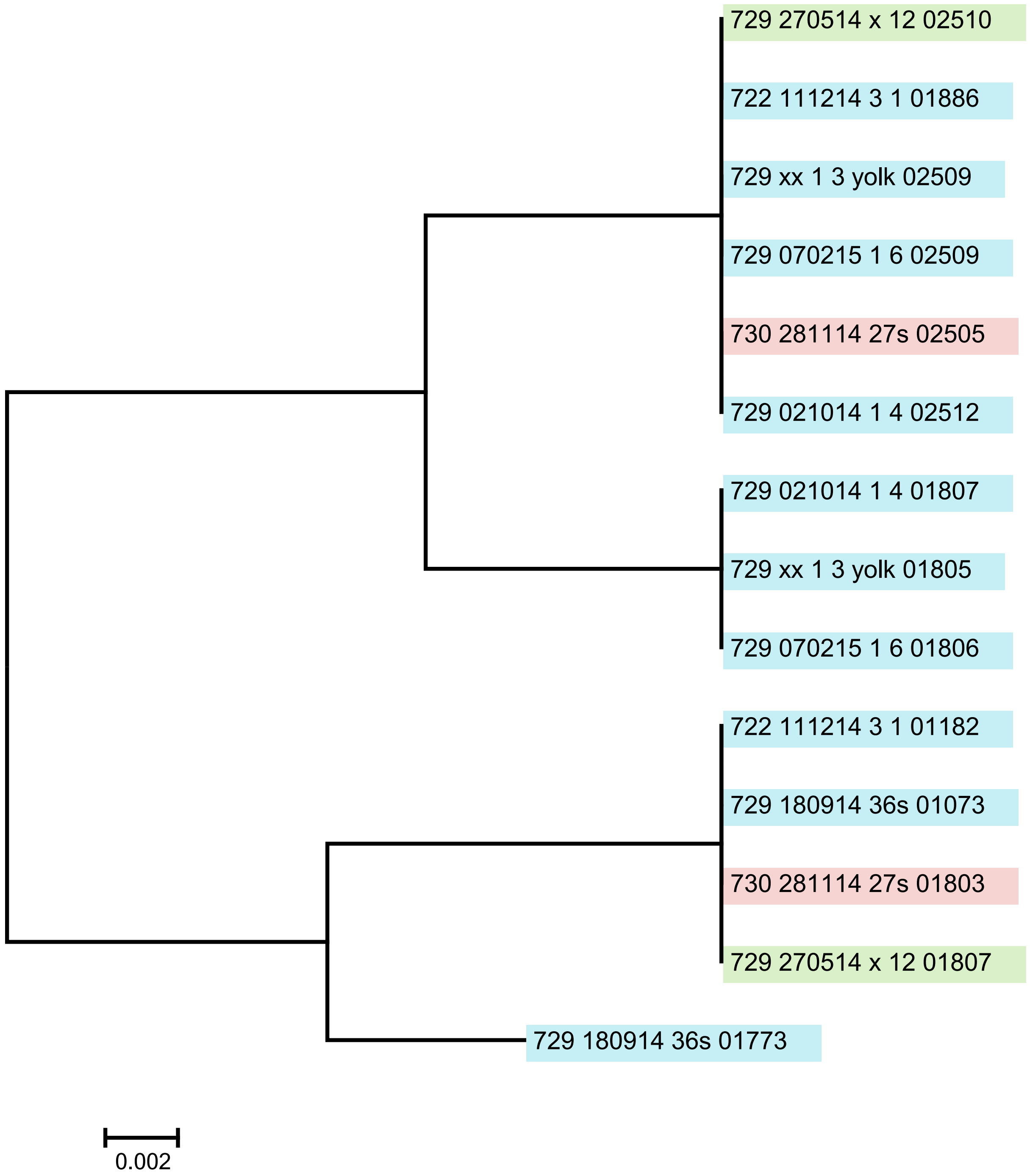

Fig. S3
ST131
hyp

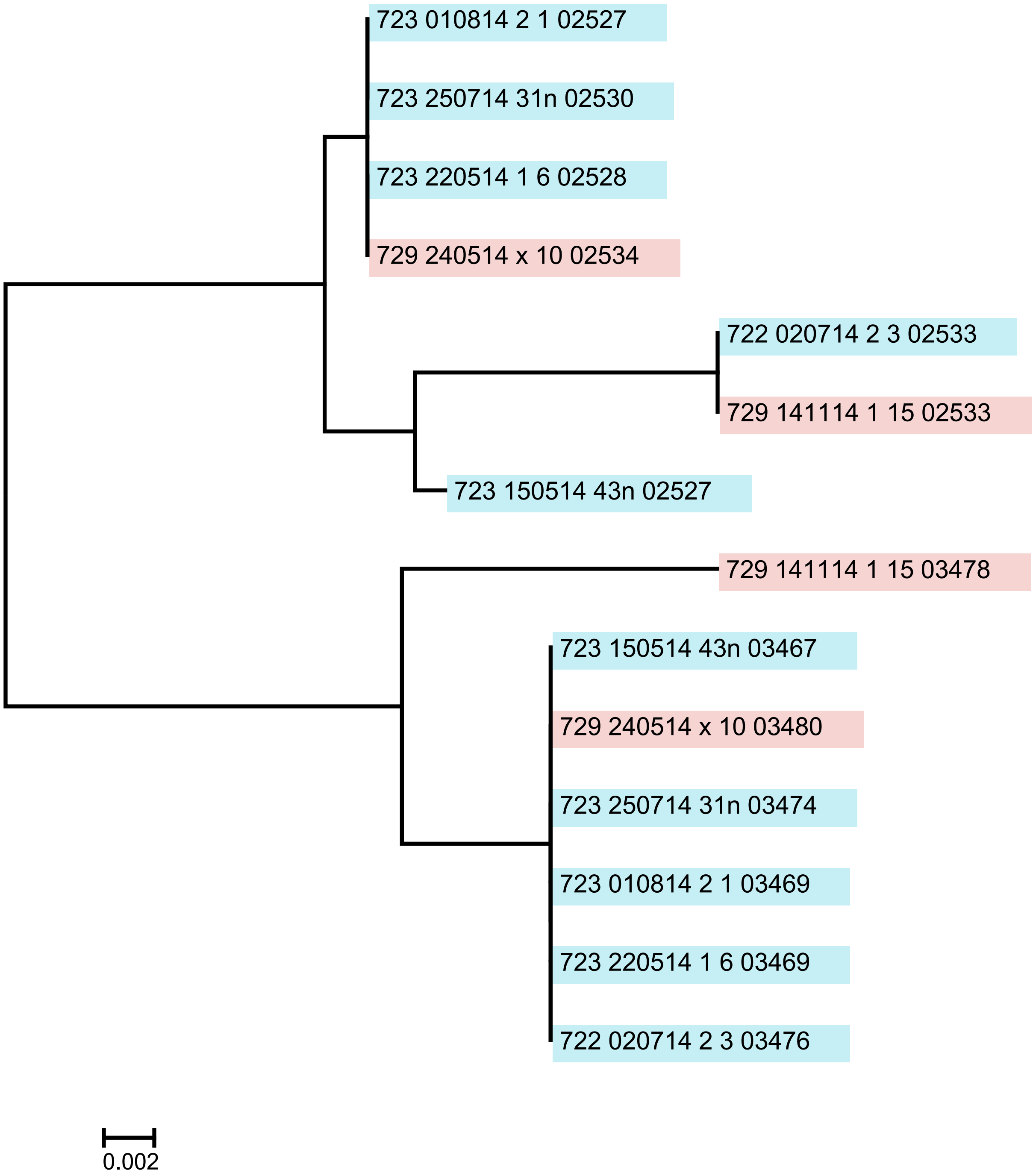

Table S1. Quality of sequencing.

ST	Strain	Illumina				Nanopore (Oxford Nanopore Technology)			
		Mean read* length (bp)	Number of reads	Total bases (Mbp)	Coverage (x)	Mean read* length (bp)	Number of reads	Total bases (Mbp)	Coverage (×)
95	729_021014_1_4	185	1,030,618	191	36	5129	137,504	706	133
	729_270514_x_12	191	1,227,066	234	44	4138	84,953	352	66
	729_xx_1_3_yolk	163	1,188,348	193	37	4260	95,064	405	76
	722_111214_3_1	175	1,431,026	250	47	4893	18,715	92	17
	729_070215_1_6	248	4,683,454	1,161	219	5133	22,897	118	22
	729_180914_36s	212	954,666	203	38	3924	125,200	491	93
	730_281114_27s	171	2,075,566	356	67	5059	23,586	119	23
	729-140714-2-2	190	814,944	160	30	ND	ND	ND	ND
	729-080814-2-9	167	1,594,368	267	50	ND	ND	ND	ND
	723-121214-2-6	187	1,049,994	197	37	ND	ND	ND	ND
131	723_010814_2_1	262	1,601,906	420	84	2710	109,803	298	60
	729_141114_1_15	176	1,186,722	209	42	3832	138,333	530	106
	723_220514_1_6_S37	207	1,237,944	257	51	3386	93,939	318	64
	722_020714_2_3	182	453,980	83	17	9039	39,799	360	72
	723_150514_43n	174	950,130	166	33	10,685	20,354	218	44
	723_250714_31n	255	837,840	214	43	4504	87,821	396	79
	729_240514_x_10	190	443,600	84	17	7748	24,398	189	38
	722-270514-2-6	181	1,148,636	208	42	ND	ND	ND	ND

* Information from SeqKit.

ND, not detected.

Table S2. Assembly parameters.

		Number of contigs		NG50 (bp)		Total length (bp)	
ST	Strain	Illumina	Hybrid	Illumina	Hybrid	Illumina	Hybrid
95	729_021014_1_4	99	5	196,246	5,232,657	5,312,171	5,437,904
	729_270514_x_12	100	4	204,287	5,232,714	5,314,927	5,435,650
	729_xx_1_3_yolk	132	5	164,290	5,232,647	5,316,910	5,437,894
	722_111214_3_1	117	7	169,295	5,133,812	5,310,534	5,432,972
	729_070215_1_6	73	5	246,237	5,232,645	5,335,036	5,437,892
	729_180914_36S	77	8	348,813	5,152,512	5,320,541	5,439,438
	730_281114_27s	99	3	204,181	5,229,948	5,305,386	5,430,207
131	723_010814_2_1	45	4	353,089	4,866,047	5,022,129	5,075,302
	729_141114_1_15	88	4	224,060	4,887,791	5,115,410	5,190,930
	723_220514_1_6_S37	50	4	353,089	4,866,598	5,022,103	5,075,810
	722_020714_2_3	109	23	138,016	500,697	5,009,973	5,079,062
	723_150514_43n	82	9	197,598	2,949,216	5,016,145	5,076,673
	723_250714_31n	48	5	320,469	4,867,625	5,023,836	5,080,162
	729_240514_x_10	150	12	67,180	3,083,037	5,113,008	5,187,247

Table S3. SNP differences among ST95-PFGE65 strains.

snp-dists 0.8.2	722_111214_3_1	729_021014_1_4	729_070215_1_6	729_080814_2_9	729_180914_36S	729_xx_1_3_yolk	723_121214_2_6	729_140714_2_2	729_270514_x_12	730_281114_27S
722_111214_3_1	0	15	12	11	12	12	29	27	25	31
729_021014_1_4	15	0	13	8	9	9	30	28	26	32
729_070215_1_6	12	13	0	9	10	10	27	25	23	29
729_080814_2_9	11	8	9	0	5	5	26	24	22	28
729_180914_36S	12	9	10	5	0	6	27	25	23	29
729_xx_1_3_yolk	12	9	10	5	6	0	27	25	23	29
723_121214_2_6	29	30	27	26	27	27	0	14	12	36
729_140714_2_2	27	28	25	24	25	25	14	0	10	34
729_270514_x_12	25	26	23	22	23	23	12	10	0	32
730_281114_27S	31	32	29	28	29	29	36	34	32	0

Table S4. SNP differences among ST131-PFGE47 strains.

snp-dists 0.8.2	722_020714_2_3	722_270514_2_6	723_220514_1_6	723_010814_2_1	723_150514_43n	723_250714_31n	729_141114_1_15	729_240514_x_10
722_020714_2_3	0	2	2	4	4	6	28	28
722_270514_2_6	2	0	2	4	4	6	28	28
723_220514_1_6	2	2	0	4	4	6	28	28
723_010814_2_1	4	4	4	0	6	8	30	30
723_150514_43n	4	4	4	6	0	8	30	30
723_250714_31n	6	6	6	8	8	0	32	32
729_141114_1_15	28	28	28	30	30	32	0	4
729_240514_x_10	28	28	28	30	30	32	4	0

Table S5. Genomes compared by hybrid assembly. Information about strains origin and associated lesions are found in TableS1.

Some information modified from Zhao et al. (1).

		Hybrid assembly		INDSC acc. no.		
Strain	PFGE-ST	Contig number	Contig length (bp)	Hybrid assembly	Short read assembly (Table S1)	Matching genomic elements
729_021014_1_4	65-95	1	5,232,657	CP160125	JBELNO01	Chromosome
		2	193,604	CP160126		Plasmid [a]
		3	6,655	CP160127		Plasmid
		4	2,676	CP160128		Plasmid
		5	2,312	CP160129		Plasmid
729_270514_x_12	65-95	1	5,232,714	CP160121	JBELNS01	Chromosome
		2	193,604	CP160122		Plasmid [a]
		3	6,655	CP160123		Plasmid
		4	2,677	CP160124		Plasmid
729_xx_1_3_yolk	65-95	1	5,232,647	CP160116	JBELNM01	Chromosome
		2	193,604	CP160117		Plasmid [a]
		3	6,655	CP160118		Plasmid
		4	2,676	CP160119		Plasmid
		5	2,312	CP160120		Plasmid
722_111214_3_1	65-95	1	5,133,812	JBMPPS01	JBELNL01	Chromosome [b]
		2	193,604			Plasmid [a]
		3	76,112			Fragment [b]
		4	11,671			Fragment [b]
		5	7,944			Fragment [b]
		6	6,655			Plasmid
		7	3,174			Plasmid
729_070215_1_6	65-95	1	5,232,645	JBMPPT01	JBELNJ01	Chromosome
		2	193,604			Plasmid [a]
		3	6,655			Plasmid
		4	2,676			Plasmid
		5	2,312			Plasmid

729_180914_36S	65-95	1	5,152,512	JBMPPU01	JBELNP01	Chromosome [b]
		2	193,604			Plasmid [a]
		3	73,951			Fragment [b]
		4	6,655			Plasmid
		5	2,728			Fragment [b]
		6	2,676			Plasmid
		7	2,312			Plasmid
730_281114_27s	65-95	1	5,229,948	JBMPPV01	JBELNN01	Chromosome
		2	193,604			Plasmid [a]
		3	6,655			Plasmid
723_250714_31n	47-131	1	4,867,625	JBMPPW01	JBELND01	Chromosome
		2	201,035			Plasmid [a]
		3	6,668			Plasmid
		4	1,552			Plasmid
723_010814_2_1	47-131	1	4,866,047	CP160112	JBELNC01 (SSQU01)	Chromosome
		2	201,035	CP160113		Plasmid [a]
		3	6,668	CP160114		Plasmid
		4	1,552	CP160115		Plasmid
729_141114_1_15	47-131	1	4,887,791	CP160108	JBELNB01	Chromosome
		2	201,054	CP160109		Plasmid [a]
		3	100,533	CP160110		Plasmid
		4	1,552	CP160111		Plasmid
723_220514_1_6	47-131	1	4,866,598	CP160104	JBELNH01	Chromosome
		2	200,992	CP160105		Plasmid [a]
		3	6,668	CP160106		Plasmid
		4	1,552	CP160107		Plasmid
722_020714_2_3	47-131	1	4,869,807	JBMPPX01		Chromosome
		2	201,035			Plasmid [a]
		3	6,668			Plasmid
		4	1552			Plasmid
723_150514_43n	47-131	1	4,867,418	JBMPPY01		Chromosome
		2	201,035			Plasmid [a]
		3	6,668			Plasmid
		4	1,552			Plasmid

729_240514_x_10	47-131	1	4,886,807	JBMPPZ01		Chromosome
		2	201,054			Plasmid [a]
		3	100,533			Plasmid
		4	3,263			Fragment [b]
		5	1,552			Plasmid
		6	1,225			Fragment [b]

[a] ColV-like

[b] no prediction of circularization

Table S6. Comparison and alignments of proteins annotated as phage tail proteins using hybrid assemblies and Prokka annotation of genomes. Information about strains origin and associated lesions are found in Table 1. Empty cells mean same sequence as the top row. Strains are colored according to clades on Fig. 1 and supplementary Fig. 1. For 729_021014_1_4 ORF 01807 a phylogenetic comparison is shown on supplementary Fig. S2.

ST95-PFGE65 strains	Query acc. no. of phage tail protein									
	YP_007112087	YP_009617242	YP_008318491	YP_008318488	YP_009617245	NP_040592				
	Subject ORF in 729_021014_1_4						Evolutionary Impact			
	02504	02512	03518	03519	01810	01807			Weighted average	
	Query in other strains						G	PAM2	G	PAM2
729_xx_1_3_yolk	N307H	NA	NA	L172V	NA	NA	32<br>68	-3<br>-2	50	-3
722_111214_3_1	N307H	NA	T56A	NA	NA	V3T, T7L, M8A, K21N, N23S, V34N, V42I	133<br>29<br>69<br>92<br>84<br>94<br>68<br>58<br>46	-6<br>-2<br>-3<br>-5<br>-4<br>-3<br>-2<br>-3<br>-2	75	-3
729_180914_36S	N307H	V3T, T7L, M8A, K21N, N23S, V34N, V42F	NA	NA	43 pos. different (or another homolog)	V3T, T7L, M8A, K21N, N23S, V34N, V42I	69<br>92<br>84<br>94<br>46<br>133<br>50	-3<br>-5<br>-4<br>-3<br>-2<br>-6<br>-6	81	-4
729_270514_x_12	G170D<br>N307H	NA	NA	NA	NA	V3T, T7L, M8A, K21N, N23S, V34N, V42I	94<br>68<br>69<br>92<br>84<br>94<br>46<br>133<br>29	-2<br>-4<br>-3<br>-5<br>-4<br>-3<br>-2<br>-6<br>-2	79	-3
730_281114_27s	G170D	NA	NA	NA	NA	V3T, T7L, M8A, K21N, N23S, V34N, V42I	94<br>69<br>92<br>84<br>94<br>46<br>133<br>29	-2<br>-3<br>-5<br>-4<br>-3<br>-2<br>-6<br>-2	80	-3

ST131-PFGE47 strain	Query acc. no. of phage tail protein							
	YP_0092119331	NP_839874	YP_0092119338					
	Subject ORF in 723_250714_31n							
	00021	00030	01486					
	Query in other strains							
723_010814_2_1	350-472 amino acid differences in length	NA	NA		ND	ND	ND	ND
723_220514_1_6	350-515 amino acid differences in protein length	NA	NA		ND	ND	ND	ND
722_020714_2_3	350-472 amino acid differences in length	NA	F40Y, I50M, E75D, R97K		22<br>10<br>45<br>26	-2<br>-3<br>-1<br>-2	26	-2
723_150514_43n	350-472 amino acid differences in length	NA	NA		ND	ND		
729_240514_x_10	350-472 amino acid differences in length	V410I	NA		29	-2	29	-2
729_141114_1_15	350-515 amino acid differences in length	V410I	NA		29	-2	29	-2

G, Grantham score (Grantham 1974); PAM2 log-odds score, point accepted mutation matrix 2 (Dayhoff et al. 1978); NA, no amino acid changes compared to top row; ND, not defined related to insertions and deletions.

Table S7. Differences in chromosomal protein sequences between seven hybrid assemblies of ST95-PFGE65. Phage related proteins excluded. NA no amino acid changes.

729_021014_1_4 as query											
**Proteins predicted from single copy genes**								Evolutionary impact			
		Strains used as subject sequences								Weighted average	
Function	Prokka CDS number used as query	729_xx_1_3_yolk	722_111214_3_1	729_070215_1_6	729_180914_36S	729_270514_x_12	730_281114_27s	G	PAM2	G	PAM2
Translation elongation factor Tu2	00111	NA	T168A	NA	NA	T168A	T168A	58	-3	58	-3
Translation elongation factor Tu1	04387	NA	NA	NA	NA	T168A	NA	58	-3	58	-3
Phosphoethanolamine trans OpgE	00350	NA	N348H,<br>I337M,<br>I415V,<br>I362V	NA	M475I,<br>E480D	N348H,<br>I337M,<br>I415V,<br>I362V	N348H,<br>I337M,<br>I415V,<br>I362V	68<br>10<br>29<br>29<br>10<br>45	-2<br>-3<br>-2<br>-2<br>-3<br>-1	32	-2
Helix-turn-helix transcriptional regulator/WYL domain-containing protein	00523	Y155C	Y155C	NA	Y155C	Y155C	Y155C	194	-4	194	-4
AroP	00812	NA	NA	NA	NA	T252P	NA	38	-4	38	-4
VasL domain-containing protein	00922	T243A	T243A	NA	T243A	T243A	T243A	58	-3	58	-3
EspC Haemoglobin binding protease hbp autotransporter	00975	T287I,<br>P332S	I418T	NA	T287I	T287I	P332S	89<br>74	-3<br>-3	82	-3
Oxogen dependent choline dehydrogenase EC 1.1.99.1	01011	NA	NA	NA	NA	T55P	NA	38	-4	38	-4
CusA	01225	V288G	NA	NA	NA	NA	A334V	109<br>64	-5<br>-3	87	-4
Nicotinate phosphoribosyl transferase EC 6.3.4.21	01571	NA	NA	NA	NA	V133G	NA	109	-5	109	-5
EfeO	01643	NA	NA	NA	NA	Q150L	Q150L	113	-4	113	-4
EntS/YbdA MFS transporter	02087	N846S,<br>N869T,<br>T504A	A357T	T765M	NA	A357T,<br>N1016K	A357T	46<br>65<br>58<br>81<br>94	-2<br>-3<br>-3<br>-4<br>-3		
Putative multidrug ATP binding permease protein	02707	NA	NA	NA	NA	GapLLLA37-40	NA	ND	ND		
JAB domain-containing protein	02801	Multicopy									
KlcA	02800	Multicopy see Fig. 3									
UTP glucanase1 phosphatase EC 2.7.7.9	02843	NA	T44I	NA	NA	T44I	T44I	89	-3		
Colonic acid WcaA	02860	NA	NA	NA	NA	L14W	L14W	61	-5		

IS31	03921	N4K,<br>R6L	NA	NA	NA	NA	NA	94<br>145	-3<br>-6	120	-5
Transposon	03922	Insert84KEAA	NA	NA	NA	Insert84KEAA	NA	ND	ND		
Hydrogenase 2 small chain	04005	NA	NA	NA	NA	I39M	I39M	10	-3	10	-3
CCA protein	04074	NA	NA	NA	NA	M234I	NA	10	-3	10	-3
RNA binding protein YhbY	04228	M32V	M32V	NA	M32V	M32V	M32V	21	-3	21	-3
General secretion pathway C	04372	NA	NA	NA	NA	R214K	NA	26	-2	26	-2
PanO	04508	D91G	D91G	NA	D91G	D91G	D91G	94	-4	94	-4
BcsE	04602	NA	NA	NA	NA	G313S	NA	56	-3	56	-3
PTS systemIIA component	04725	NA	NA	NA	NA	A72D	NA	126	-4	126	-4
**Proteins predicted from multi copy genes**											
Hypothetic	00307/04763	E30G	E30G	NA	E30G	NA	E30G	98	-4	98	-4
Colicin I receptor	00313/03923	Q637L	Q637L, V313I	NA	Q637L	NA	NA	113<br>29	-4<br>-2	71	-3
Helicase putative	00351<br>03887	R630S	T141I,<br>T144S,<br>V153I,<br>E165A,<br>I738V,<br>Y803F	NA	T141I,<br>T144S,<br>V153I,<br>E165A,<br>R630S,<br>Y803F,<br>K1048R,<br>K1081V,<br>D1093S	T141I,<br>T144S,<br>V153I,<br>E165A,<br>R630S,<br>Y803F	T141I,<br>T144S,<br>V153I,<br>E165A,<br>I738V,<br>Y803F	89<br>58<br>29<br>107<br>89<br>22<br>110<br>26<br>97<br>65	-3<br>-2<br>-2<br>-3<br>-3<br>-2<br>-3<br>-2<br>-6<br>-4	69	-3
IS1 family transposase IS1A	00495	A68V	NA	A68V	A68V	A68V 8 copies intra	A68V	64	-3	64	-3
Hypothetical protein	00528	NA	NA	NA	NA	H16R 2 copies	NA	29	-3	29	-3
IS1 family transposase IS1A	02762	A68V 8 copies	5 copies	copies	NA	A68V 8 copies	4 copies	64	-3	64	-3
Transposon	03885	NA	NA	NA	E264K,<br>S248A, S255A<br>2 copies	E264K,<br>S248A,<br>S255A 2 copies	NA	54	-4	54	-4
YafY	03932	Y78C	NA	NA	NA	Y78C2 copies	NA	194	-4	194	-4
InsD, two copies identical protein sequence	02841<br>05070	A223T,<br>T227P	NA	NA	NA	NA	NA	58<br>38	-3<br>-4	48	-4
IS1A transposase up to 8 copies all identical protein sequence	04731	A68V	NA	NA	A68V	NA	NA	64	-3	64	-3

G, Grantham score (Grantham 1974); PAM2 log-odds score, point accepted mutation matrix 2 (Dayhoff et al. 1978); NA, no amino acid changes compared to top row; ND, not defined related to insertions and deletions.

Table S8. Differences in chromosomal protein sequences between seven hybrid assemblies of ST131-PFGE47. Phage related proteins excluded. NA no amino acid changes.

723_010814_2_1 as query											
**Proteins predicted from single copy genes**								Evolutionary impact			
		Strains used as subject sequences								Weighted average	
Function	Prokka CDS number used as query	729_141114_1_15	729_240514_x_10	723_220514_1_6	723_250714_31n	722_020714_2_3	723_150514_43n	G	PAM 2		
MdtN	00235	A189V	A189V	NA	NA	NA	NA	64	-3	64	-3
ATP transporter	00241	Q369K	Q369K	Q369K	Q369K	Q369K	Q369K	53	-3	53	-3
RpiR	00243	R104P	R104P	NA	NA	NA	NA	103	-4	103	-4
PTS system	00357	C347W	NA	NA	NA	NA	NA	215	-11	215	-11
TAM	00392	P1098Q	NA	NA	NA	NA	NA	76	-3	76	-3
ISKra4 transposase IScep1	00461	F75L	NA	NA	NA	NA	NA	22	-3	22	-3
TctB	00635	V81L	V81L	NA	NA	NA	NA	32	-3	32	-3
1-deoxy-D-xylose-5-phophatre-reductoisomerase	00749	NA	NA	S16C	NA	NA	NA	112	-3	112	-3
FliO, flagellum specific ATP synthase	00815	G249R	G249R	NA	NA	NA	NA	125	-6	125	-6
SbcCD	00964	A522V	A522V	NA	NA	NA	NA	64	-3	64	-3
Sialidase family protein	01224	G335S	G335S	NA	NA	NA	NA	56	-3	56	-3
AbrB	01256	V304A	V304A	NA	NA	NA	NA	64	-3	64	-3
CydD	01435	A190T	A190T	NA	NA	NA	NA	58	-3	58	-3
Transposase	01805	K66I	K66I	NA	NA	NA	NA	102	-3	102	-3
SapA	01866	E164D	E164D	NA	NA	NA	NA	45	-1	45	-1
Large exoprotein EntS/YbdQA MFS transporter	01924<br>Deletion of 293 amino acids<br>https://www.elgiganten.dk/product/computer-kontor/computere/stationar-pc/lenovo-ideacentre-08irh9-i5-138512-stationar-computer/913589#specifications	T766K, V809I, K821N, T962K, K1019N, E1208D	T766K, V809I, K821N<br>K919N, T962K, K1019N,<br>Insertion 97 aa	K821N	K570T, K821N<br>Divergent sequence in 460-478	K570T, K821N	K570T, K821N	78<br>45<br>94<br>78<br>29<br>94<br>78<br>94	-4<br>-1<br>-3<br>-4<br>-2<br>-3<br>-4<br>-3	74	-3
YeaD putative glucose 6-phosphate 1 epimerase	02228	A53V	A53V	NA	NA	NA	NA	64	-3	64	-3
2-succinyl-5 enol pyravyl 6-hydroxy 3 cyclohexane 1-carbopxyl synthase	02797	N235K	N235K	NA	NA	NA	NA	94	-3	94	-3

NADH-quinone-oxidoreductase subunit F	02813	K80E	K80E	NA	NA	NA	NA	56	-4	56	-4
YfcP putative fimbria like protein	02862	R58C	R58C	NA	NA	NA	NA	180	-5	180	-5
flavodoxin	03019	A56T	A56T	NA	NA	NA	NA	58	-3	58	-3
Penicillin-binding protein 1C	03023	D389A	D389A	NA	NA	NA	NA	12	-4	12	-4
YphB	03042	S239P	S239P	NA	NA	NA	NA	74	-3	74	-3
GhxQ	03353	F76K	F76K	NA	NA	NA	NA	102	-11	102	-<br>11
Small-conductance mechanosensitive channel	03390	T19A	T19A	NA	NA	NA	NA	58	-3	58	-3
DctD	03451	L308P	L308P	NA	NA	NA	NA	98	-5	98	-5
Hyp	03469	V18I, V25L, K33Q, Q48R, S59P, A139S, H155Y	NA	NA	NA	NA	NA	43<br>29<br>32<br>53<br>74<br>99<br>83	-3<br>-2<br>-3<br>-3<br>-3<br>-3<br>-4	59	-3
	03514	R345G	R345G	NA	NA	NA	NA	125	-6	125	-6
TdcG	03663	P84S	P84S	NA	NA	NA	NA	74	-3	74	-3
tRNA pseudouridine-synthase B	03710	G8-, R9-	G8-, R9-	NA	NA	NA	NA	ND	ND		
CsrD	03787	F292S	F292S	NA	NA	F292S	NA	155	-5	155	-5
Ribosome associated	03938	N37H	N37H	N37H	N37H	N37H	N37H	68	-2	68	-2
Glucose 6-phosphate adenylyl transferase	03969	D239N	D239N	NA	NA	NA	K195N	23,<br>94	-2,<br>-3	23,<br>94	-2,<br>-3
TusA	04007	R50H	R50H	NA	NA	NA	NA	29	-3	29	-3
Hypothetic protein	04311	N166H	N166H	NA	NA	NA	NA	68	-2	68	-2
Carbohydrate acetylesterase/feruloy l esterase	04370	F16I	F16I	NA	NA	NA	NA	21	-3	21	-3
**Proteins predicted from multi copy genes**											
IIS3 family transposase IS1397	02468/0230	NA	L33Q, A37E, NA	L33Q, A37E	L33Q, A37E	NA	L33Q, A37E	107<br>113	-3<br>-4	110	-4
Hyp see Fig. S3	02527	V18I, V25L, K33Q, Q48R, S59P, A139S, H155Y	2 copies	NA	NA	NA	NA	32<br>29<br>74<br>53<br>43<br>83<br>99	-3<br>-2<br>-3<br>-3<br>-3<br>-4<br>-3	110	-4
Transposase	02468/02303	NA	L33Q, A37E, NA	L33Q, A37E	L33Q, A37E	NA	L33Q, A37E	113<br>,	-4,<br>-3	110	-4

								107			

G, Grantham score (Grantham 1974); PAM2 log-odds score, point accepted mutation matrix 2 (Dayhoff et al. 1978); NA, no amino acid changes compared to top row; ND, not defined related to insertions and deletions.
